# Bacteriophage P22 SieA-mediated superinfection exclusion

**DOI:** 10.1128/mbio.02169-23

**Published:** 2024-01-18

**Authors:** Justin C. Leavitt, Brianna M. Woodbury, Eddie B. Gilcrease, Charles M. Bridges, Carolyn M. Teschke, Sherwood R. Casjens

**Affiliations:** 1 School of Biological Sciences, University of Utah, Salt Lake City, Utah, USA; 2 Department of Molecular and Cell Biology, University of Connecticut, Storrs, Connecticut, USA; 3 Division of Microbiology and Immunology, Pathology Department, University of Utah School of Medicine, Salt Lake City, Utah, USA; 4 Department of Chemistry, University of Connecticut, Storrs, Connecticut, USA; Massachusetts Institute of Technology, Cambridge, Massachusetts, USA

**Keywords:** P22, bacteriophage, superinfection exclusion, ejection proteins, SieA

## Abstract

Many temperate phages encode prophage-expressed functions that interfere with superinfection of the host bacterium by external phages. *Salmonella* phage P22 has four such systems that are expressed from the prophage in a lysogen that are encoded by the *c2* (repressor), *gtrABC*, *sieA*, and *sieB* genes. Here we report that the P22-encoded SieA protein is necessary and sufficient for exclusion by the SieA system and that it is an inner membrane protein that blocks DNA injection by P22 and its relatives, but has no effect on infection by other tailed phage types. The P22 virion injects its DNA through the host cell membranes and periplasm via a conduit assembled from three “ejection proteins” after their release from the virion. Phage P22 mutants that overcome the SieA block were isolated, and they have amino acid changes in the C-terminal regions of the gene *16* and *20* encoded ejection proteins. Three different single-amino acid changes in these proteins are required to obtain nearly full resistance to SieA. Hybrid P22 phages that have phage HK620 ejection protein genes are also partially resistant to SieA. There are three sequence types of extant phage-encoded SieA proteins that are less than 30% identical to one another, yet comparison of two of these types found no differences in phage target specificity. Our data strongly suggest a model in which the inner membrane protein SieA interferes with the assembly or function of the periplasmic gp20 and membrane-bound gp16 DNA delivery conduit.

**IMPORTANCE:**

The ongoing evolutionary battle between bacteria and the viruses that infect them is a critical feature of bacterial ecology on Earth. Viruses can kill bacteria by infecting them. However, when their chromosomes are integrated into a bacterial genome as a prophage, viruses can also protect the host bacterium by expressing genes whose products defend against infection by other viruses. This defense property is called “superinfection exclusion.” A significant fraction of bacteria harbor prophages that encode such protective systems, and there are many different molecular strategies by which superinfection exclusion is mediated. This report is the first to describe the mechanism by which bacteriophage P22 SieA superinfection exclusion protein protects its host bacterium from infection by other P22-like phages. The P22 prophage-encoded inner membrane SieA protein prevents infection by blocking transport of superinfecting phage DNA across the inner membrane during injection.

## INTRODUCTION

Temperate tailed bacteriophages can use one of two strategies for replication: a lytic cycle, which is a productive infection that releases progeny virions through host cell lysis, or a lysogenic cycle, which establishes a prophage state where the integrated phage DNA replicates in synchrony with the host chromosome without major detriment to the host cell. Although prophages do not express the genes required for the lytic cycle, they do express a few genes whose products often modify their bacterial host. A significant fraction of such “lysogenic conversion” genes act to protect the host bacterium from infection by other phages, which is called superinfection exclusion.

Prophage-expressed superinfection exclusion systems have been studied in numerous phages, and they include systems that block infecting phages at various stages of the lytic cycle (reviewed by references 1–3). Some prophages inhibit DNA delivery into host cells by expressing proteins that modify bacterial surface phage receptors and thus prevent the adsorption of superinfecting phages. These include enzymes that alter surface polysaccharides (4, 5) and proteins that interfere with the accessibility of outer membrane proteins (6, 7). Other superinfection exclusion systems block phage development after the initial adsorption event. For example, prophage-encoded proteins can block DNA injection by unknown mechanisms (e.g., *Escherichia coli* phage HK97 gp15 [8], *Streptococcus thermophilus* phage TP-J34 Ltp protein [9], and *Lactococcus lactis* phage Tuc2009 Sie_2009_ protein [10]). Yet other exclusion systems interfere with incoming phages at later stages of the replication cycle; for instance, abortive infection systems cause replication failure in mid-lytic growth. In such cases, the infected cell dies but does not release progeny viruses, thus preventing their spread (reviewed by reference [11).

*Salmonella enterica* phage P22 prophages interfere with superinfecting phages in at least four different ways. These include (i) the prophage C2 repressor protein that prevents lytic gene expression by homo-immune phages (12–14); (ii) the three GtrABC proteins that alter the *S. enterica* Typhimurium O-antigen surface polysaccharide to block adsorption by phages that utilize it as a receptor (5, 15, 16); and two superinfection exclusion systems mediated by the less well-understood (iii) *sieA* and (iv) *sieB* genes (17–19). The SieB exclusion system causes cellular macromolecular synthesis to cease midway through the lytic cycle during superinfection by P22-like phages but not by P22 itself (19–21). The SieA protein blocks superinfection by P22-like phages (including P22 itself) at an early stage of infection (19, 22–24).

Previous studies have shown that a *sieA*^+^ P22 prophage does not affect the adsorption of superinfecting P22 phage virions (18, 23, 24) or the production of progeny after P22 prophage induction (24). SieA exclusion is not specific for the type of DNA in the infecting virion since generalized transduction by phage P22 is also greatly lowered by a *sieA^+^* 22 prophage in the recipient cell (19, 22–24). In a *sieA^+^* lysogen, superinfecting P22 DNA is not cleaved by cytoplasmic host restriction endonucleases, and superinfecting P22 genes are not expressed (20). These findings suggest that the *sieA* block occurs early in the infection cycle after adsorption has occurred, and the current hypothesis is that the SieA protein blocks entry of superinfecting phage DNA into the target cell.

Injection of DNA from short-tailed phage virions, like those of P22, into target cells is mediated by a transperiplasm conduit assembled from ejection proteins (E-proteins) that are released from the infecting virions (25–30). In phage P22, this conduit is built from the protein products of phage genes *7*, *16*, and *20* (28, 31–35). *Salmonella enterica* phage P22 SieA protein is the only system studied to date that is thought to block DNA injection by superinfecting short-tailed phages. In this report, we examine phage P22 *sieA*-mediated superinfection exclusion in light of this DNA injection mechanism in more detail.

## RESULTS

### The phage P22 *sieA* gene is sufficient to block phage DNA injection

**Fig 1:**
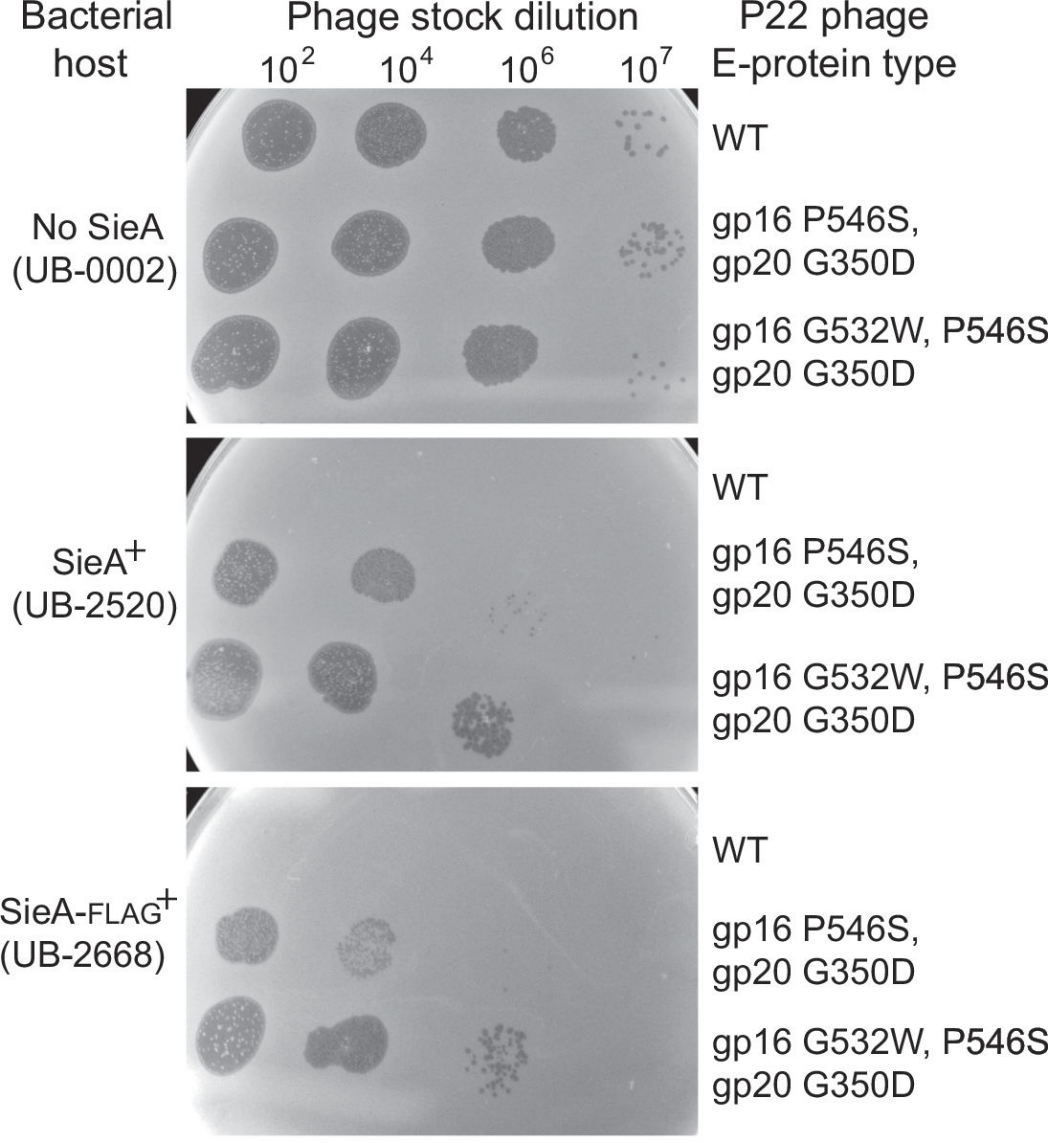
SieA superinfection exclusion. Soft agar containing *sieA^+^* or *sieA^–^ S. enterica* lawn cells (indicated with relevant genotype on the left) was allowed to solidify on L broth plates, and 10-μL spots of parental “wild-type” (WT) phage (P22 UC-0937), as well as isogenic double-mutant (P22 UC-0972) and isogenic triple-mutant (P22 UC-0979) phages that overcome *sieA* exclusion, were applied (indicated on the right). See text and Table 1 for descriptions and complete genotypes of these phages and lawn cells. The plates were incubated overnight at 37°C. Phage stocks were about 10^10^ PFU/mL, and they were diluted by the factors shown above. Ten microliters of undiluted P22 (UC-0937) phage stock also showed no plaques on *sieA^+^* host (UB-2520).

Prophages with a mutant *sieA* gene fail to exclude P22 (18, 24), and a high copy number plasmid carrying the P22 *sieA*, *orf59a*, and *mnt* genes conveys strong resistance to P22 infection (36). In order to determine whether the *sieA* gene alone in low copy number is sufficient for superinfection exclusion, we inserted it into the chromosome of *S. enterica* LT2 strain UB-0002 by recombineering (see Materials and Methods; bacterial and phage strains used are listed in Table 1). In the resulting strain (UB-2520), the inserted *sieA* gene replaces the *galK* gene in the same transcriptional orientation as the *gal* operon. The inserted DNA includes only the *sieA* gene and the putative P*_sieA_* promoter (36) with 85 bp upstream of the *sieA* start codon and 22 bp downstream of the stop codon. Since there was no galactose in the growth medium, *sieA* expression was likely governed by P*_sieA_* in this strain. Fig. S1 shows that the chromosomal *sieA* gene has little, if any, effect on cell growth at 37°C in rich medium. This *sieA^+^* was not lysed by P22 infection in liquid culture at a multiplicity of infection (MOI) of 7, and the inserted *sieA* gene lowered the plaque-forming ability of P22 by at least seven orders of magnitude (Fig. 1). We note that loss of host *galK* function alone in the isogenic *sieA^–^* strain (UB-2666) does not affect P22 infection. Thus, the *sieA* gene in one copy per *Salmonella* genome is sufficient to mediate robust superinfection exclusion.

**TABLE 1:** Bacteria and bacteriophage strains used in this study

Name	Genotype^*a*^	Reference
*Salmonella enterica* serovar Typhimurium LT2		
UB-0002	(DB7004) *leuA am*414, *supE;* gift from J. King	(37)
UB-0020	(MS1868) *leuA am*414, ∆Fels2, r^–^, m^–^, sup**°**; gift from M. Susskind	(38)
UB-1936	UB-0020 *galK::TetRA*-1 (P22 *13^–^ am*H101, *15*^–^ ∆sc302::Kan^R^, *sieA*^–^ ∆1, *20*^–^ amL100)	This report
UB-2158	UB-0020 *galK::TetRA*-1 (P22 *c1*^7^, *13*^*–*^*am*H101, orf25::Cam^R^-EG1, *sieA*^–^∆1 = P22 UC-0937)	This report
UB-2232	(SDT2739) ∆Fels-1, ∆Fels-2, ∆Gifsy-1, ∆Gifsy-2; gift from A. Segall	This report
UB-2285	UB-2232 *galK::TetRA*-1 (P22 UC-0937 with *20*^–^∆−1)	(39)
UB-2288	UB-2232 *galK::TetRA*-1 (P22 UC-0937 with *16*^*–*^∆−1)	(39)
UB-2289	UB-2232 *galK::TetRA*-1 (P22 UC-0937 with *7*^–^∆−1)	(39)
UB-2343	UB-0020 *galK::TetRA*-1 (P22 UC-0937 with gp16 P546S)^*c*^	This report
UB-2349	UB-0020 *galK::TetRA*-1 (P22 UC-0937 with Sf6 genes *7*, *20*, and *16*)^*b*^	This report
UB-2416	UB-0020 *galK::TetRA*-1 (P22 UC-0937 with Sf6 gene *7*)^*b*^	This report
UB-2417	UB-0020 *galK::TetRA*-1 (P22 UC-0937 with Sf6 gene *20*)^*b*^	This report
UB-2418	UB-0020 *galK::TetRA*-1 (P22 UC-0937 with Sf6 gene *16*)^*b*^	This report
UB-2430	UB-0020 *galK::TetRA*-1 (P22 UC-0937 with gp16 P546S, gp20 G350D = UC-0972)^*c*^	This report
UB-2461	UB-0020 *galK::TetRA*-1 (P22 S1; see Table S1)	This report
UB-2464	UB-0020 *galK::TetRA*-1 (P22 UC-0937 with L genes *7*, *20*, and *16*)^*b*^	This report
UB-2474	UB-0020 *galK::TetRA*-1 (P22 UC-0937 with gp16 P546S, gp20 T341I)^*c*^	This report
UB-2475	UB-0020 *galK::TetRA*-1 (P22 UC-0937 with gp16 P546S, gp20 T338I)^*c*^	This report
UB-2476	UB-0020 *galK::TetRA*-1 (P22 UC-0937 with gp16 P546S, gp20 T348I)^*c*^	This report
UB-2520	UB-0002 *galK::P22 sieA*	This report
UB-2537	UB-0020 *galK::TetRA*-1 (P22 UC-0937 with HK620 genes *7*, *20*, and *16*)^*b*^	This report
UB-2614	UB-0020 *galK::TetRA*-1 (P22 UC-0937 with CUS-3 genes *7*, *20*, and *16*)^*b*^	This report
UB-2621	UB-0021 *galK::HS1 sieA*	This report
UB-2631	UB-0020 *galK::TetRA*-1 (P22 UC-0937 with gp16 P546S and G532W, gp20 T341I)^*c*^	This report
UB-2634	UB-0020 *galK::TetRA*-1 (P22 UC-0937 with gp16 G532W, gp20 G350D)^*c*^	This report
UB-2653	UB-0020 *galK::TetRA*-1 (P22 UC-0937 with gp16 P546S and G532W, gp20 G350D)^*c*^	This report
UB-2666	UB-0002 *galK*Δ-1 (*galK* gene neatly deleted)	This report
UB-2668	UB-0002 *galK::P22 sieA* C-terminal SieA FLAG-tag	This report
UB-2669	UB-0020 *galK::TetRA*-1 (P22 UC-0937 with Sf6 genes *16* and *20*)^*b*^	This report
UB-2671	UB-0020 *ΔyajC::*Kan^R^	This report
UB-2676	UB-0002 *galK::P22 sieA* (P22 UC-0937)	This report
*Escherichia coli*		
UB-0049	(K12 NF1829) *araD*^–^139, ∆7679(*araABOIC*^–^, *leu*^–^), *galUK*^–^, *lac*^–^∆X74, *rspL*^–^, thi^–^ /F^’^ *lac* Iq1, *lac Z*::Tn5(Kan^R^), *lacY*^+^	(40)
UB-1702	Host for phage HK620; gift of strain 2158 from A. J. Clark	(41)
UB-1732	Strain HS, gift from J. Nataro	(42)
UB-1957	Host for phage CUS-3, gift of strain EV36 from E. Vimr	(43)
UB-2478	UB-1957 *galK::P22 sieA*	This report
UB-2558	UB-1702 *galK::P22 sieA-Kan*^R *d*^	This report
Bacteriophages		
9NA	*Salmonella* phage, prototype member of 9NA-like cluster, gift from C. Miller	(44)
CUS-3	P22-like *E. coli* phage, gift from E. Vimr	(43)
Det7	*Salmonella* phage, member of Vi01-like phage cluster, gift from S. Miller	(45)
Felix-O1	*Salmonella* phage, member of Felix-O1-like cluster, gift from D. Botstein	(46)
HK620	P22-like *E. coli* phage, gift from A. J. Clark	(41)
L	P22-like *Salmonella* phage, gift from D. Botstein	(47)
LP7	P22-like *Salmonella* phage, gift from H. Schmieger	(48)
MG40	P22-like *Salmonella* phage, gift from D. Botstein	(49)
MG178	P22-like *Salmonella* phage, gift from D. Botstein	(50)
Sf6	P22-like *Shigella* phage, gift from R. Morona	(51)
SP6	*Salmonella* phage, member of T7-like supercluster, gift from I. Molineux	(52)
Utah	*Salmonella* phage, member of chi-like cluster	(53)
UC-0011	P22 *c1*^*7*^, *13*^*–*^*am*H101; gift from J. King	(33)
UC-0763	P22 *c1^7^*	(54)
UC-0937	P22 *c1^7^*, *13*^*–*^*am*H101, orf25::Cam^R^-EG1, *sieA*^–^∆1	This report
UC-0938	P22 *13*^*–*^*am*H101, orf25::Cam^R^-EG1, *sieA*^–^∆1	This report
UB-0972	P22 UC-0937 with gp16 P546S, gp20 G350D	This report
UC-0979	P22 UC-0937 with gp16 P546S & G532W, gp20 G350D	This report
UC-0980	P22 UC-0937 with gp16 P546S & G532W, gp20 T341I	This report
UC-0838	P22 *20*^–^*am*H1032, gift from David Botstein	(55)

^*a*^ Bacterial strain names UB-xxxx at the left in the middle column indicate that the bacterial strain also carries the chromosomal alleles of that strain.

^*b*^ P22 genes *7*, *16*, and/or *20* were neatly replaced by their homologs from the indicated phages by recombineering (we use the P22 gene names here, although each of the other phages uses different names). The phage L replacement is an exception in which the replacement also included the region between phage L genes *16* and *9*. Their structures were confirmed by whole-genome sequencing.

^*c*^ The indicated single-amino acid changing point mutations (see text and Table S1) were placed in P22 prophage UC-0937 of strain UB-2158 by recombineering (see Materials and Methods).

^*d*^ Due to difficulties with *galK* recombineering in this host, a kanamycin resistance cassette was joined to *sieA* gene-containing DNA, and the gene pair was inserted by homologous recombination.

To show that under these conditions the superinfection block is very early in infection, we compared the frequency of lysogen formation by P22 in isogenic *sieA^+^* and *sieA^–^* host strains. Lysogenization should be a more accurate test for SieA exclusion than plaque formation since a rare successful single DNA entry event can give rise to a lysogen cell, whereas plaque formation requires successful infection in several successive rounds of infection. Isogenic cells with and without a chromosomal *sieA^+^* gene (UB-2520 and UB-2666, respectively) were infected by P22 UC-0938 at an MOI of 25 plaque-forming units (PFU)/cell at 37°C in rich medium. This lysogen-forming *c^+^* P22 strain carries a Cam^R^ chloramphenicol resistance cassette that allows selection for lysogens. It also carries the *13^–^am*H101 allele, which blocks phage-mediated lysis. However, infected cells can be lysed chemically with chloroform. The resulting number of lysogen (chloramphenicol-resistant) colonies after plating 30 min post-infection indicated a frequency of lysogen formation of about 25% for cells that do not carry a *sieA* gene, but the frequency was 10^5^-fold lower in the *sieA^+^* cells. Thus, SieA has a strong negative effect on lysogen formation, which confirms that the block is indeed early in infection.

**Fig 2:**
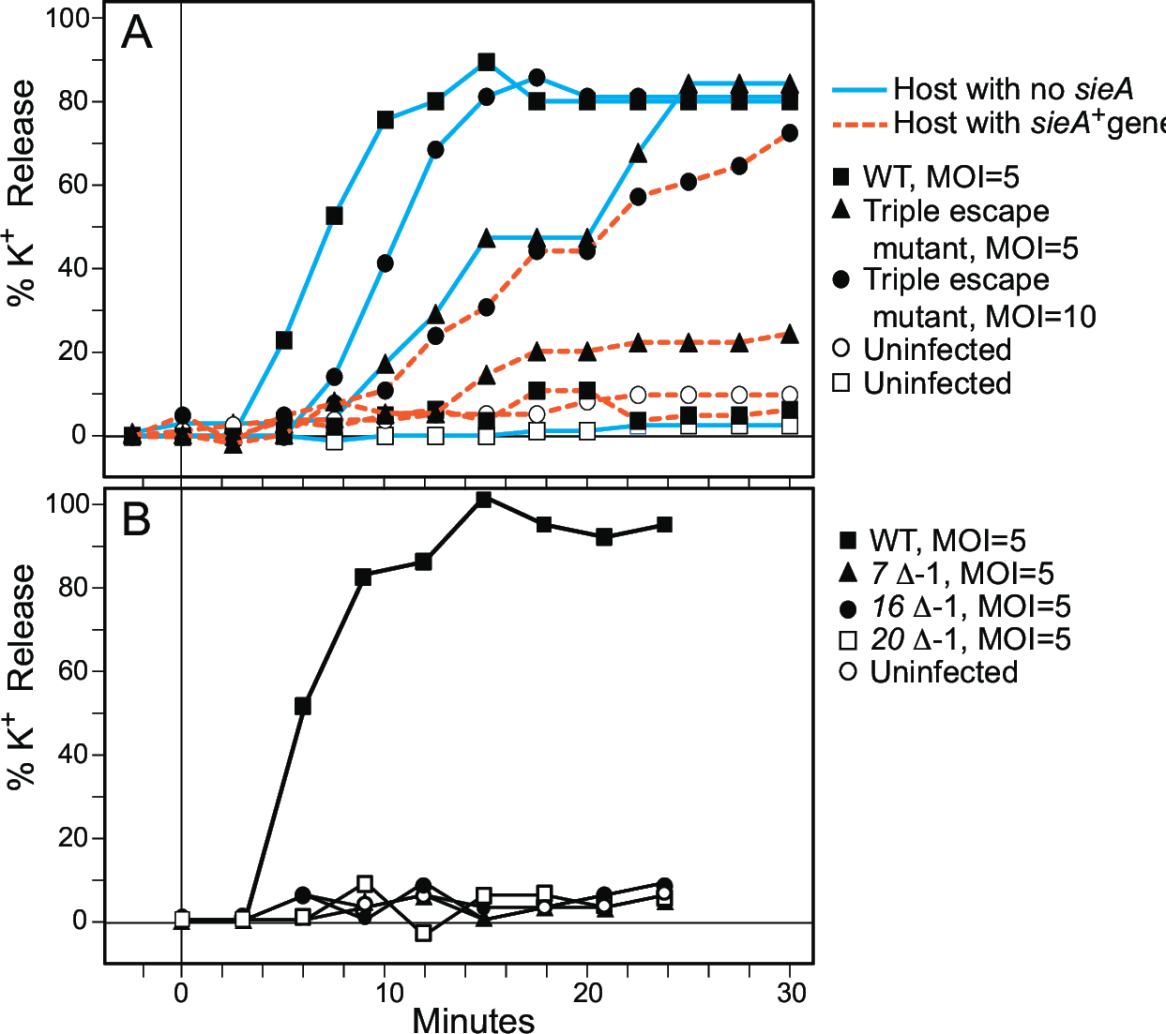
Potassium ion release after P22 infection. *S. enterica* strains growing in log phase were infected with P22 at 37°C, and K^+^ measurements (expressed as percent released relative to total K^+^ released after boiling for 10 min) were performed as described (57). (A) Potassium ion release by infectious P22 virions. The upper key gives the infections that were analyzed. The host with no *sieA* gene was UB-0002 and with a *sieA^+^* gene was UB-2520. The infecting phages were as follows: WT P22 (UC-0937), triple SieA escape mutant P22 *16* G532W, *16* P546S, and *20* G350D (UC-0979). (B) Lack of potassium ion release by P22 particles lacking E-proteins. The host with no *sieA* gene (UB-0002) was infected as indicated in the lower key with “WT” P22 (UC-0937) or by virion-like particles whose genomes carry a deletion of one of the E-protein genes and which do not make plaques. The latter defective particles were produced by induction of isogenic *7*^–^D-1, *16*^–^D-1, and *20*^–^D-1 prophages in strains UB-2289, UB-2288, and UB-2285, respectively. The virions and virion-like particles were CsCl gradient purified, and relative particle concentrations were measured by quantitating the amount of coat protein in Coomassie brilliant blue-stained SDS-PAGE gels.

Potassium ion (K^+^) release correlates very well with the successful delivery of DNA into the cytoplasm of target cells by a number of tailed phages including P22 (8, 56, 57). Although the mechanism of K^+^ release upon infection is not fully understood, presumably K^+^ escapes through the same channel with which the DNA enters the cell. Therefore, to test the ability of phage P22 to inject its DNA into *Salmonella* cells expressing SieA, a potassium selective electrode was used to measure K^+^ ion release into the surrounding medium from P22 infected cells. Figure 2A shows that P22 causes very little, if any, K^+^ release from the *sieA^+^* strain, while nearly all of the cellular K^+^ is released from the isogenic *sieA^–^* strain, suggesting that DNA injection *per se* is blocked by SieA.

Finally, when cultures of isogenic *Salmonella* strains carrying *sieA^–^* P22 prophages with and without an ectopic *sieA^+^* gene (UB-2676 and UB-2158, respectively) were induced by addition of mitomycin C to 0.1 μg/mL, they gave indistinguishable phage yields of about 10^10^ PFU/mL (data not shown). This result indicates that post-induction lytic growth and generation of phage progeny is not affected by SieA. We conclude from the above results that SieA alone blocks injection of P22 DNA into *Salmonella* cells and has no other additional effect on P22 growth.

### The presence of SieA and absence of E-proteins have similar phenotypes

The phenotype of the P22 SieA block is similar to that of P22 virion-like particles produced by gene *7, 16*, or *20* mutants. The protein products of these genes are released from the virion during DNA delivery and form a transmembrane conduit that extends from the virion to the cytoplasm (above). Mutations that inactivate genes *7*, *16*, or *20* result in the formation of virions that lack the affected protein and that do not deliver their DNA into target cells properly (31–34).

Figure 2B shows that infection by E-protein-deficient particles fail to release K^+^, which is similar to the infection of *sieA^+^* by wild-type P22 (above). Furthermore, experiments using 7 or 30 particles per cell also showed no measurable K^+^ release. Ejection of gp16 and gp20 is each independent of the presence of the other two E-proteins, and gp7 release is independent of the presence of gp20. However, gp7 is not released from particles that lack gp16 (32). Thus, release of gp20 through infection with *16^–^* phages, gp7 + gp16 by *20^–^* phages, or gp16 + gp20 by *7^–^* phages is not sufficient for release of K^+^ from the cell. In other words, all three E-proteins are required for K^+^ release and for DNA injection. The similar K^+^ release and DNA injection phenotypes that result from the SieA block and from the absence of any of the E-proteins make the idea that SieA may interfere with E-protein function during DNA injection an attractive hypothesis.

### SieA is an inner membrane protein

**Fig 3:**
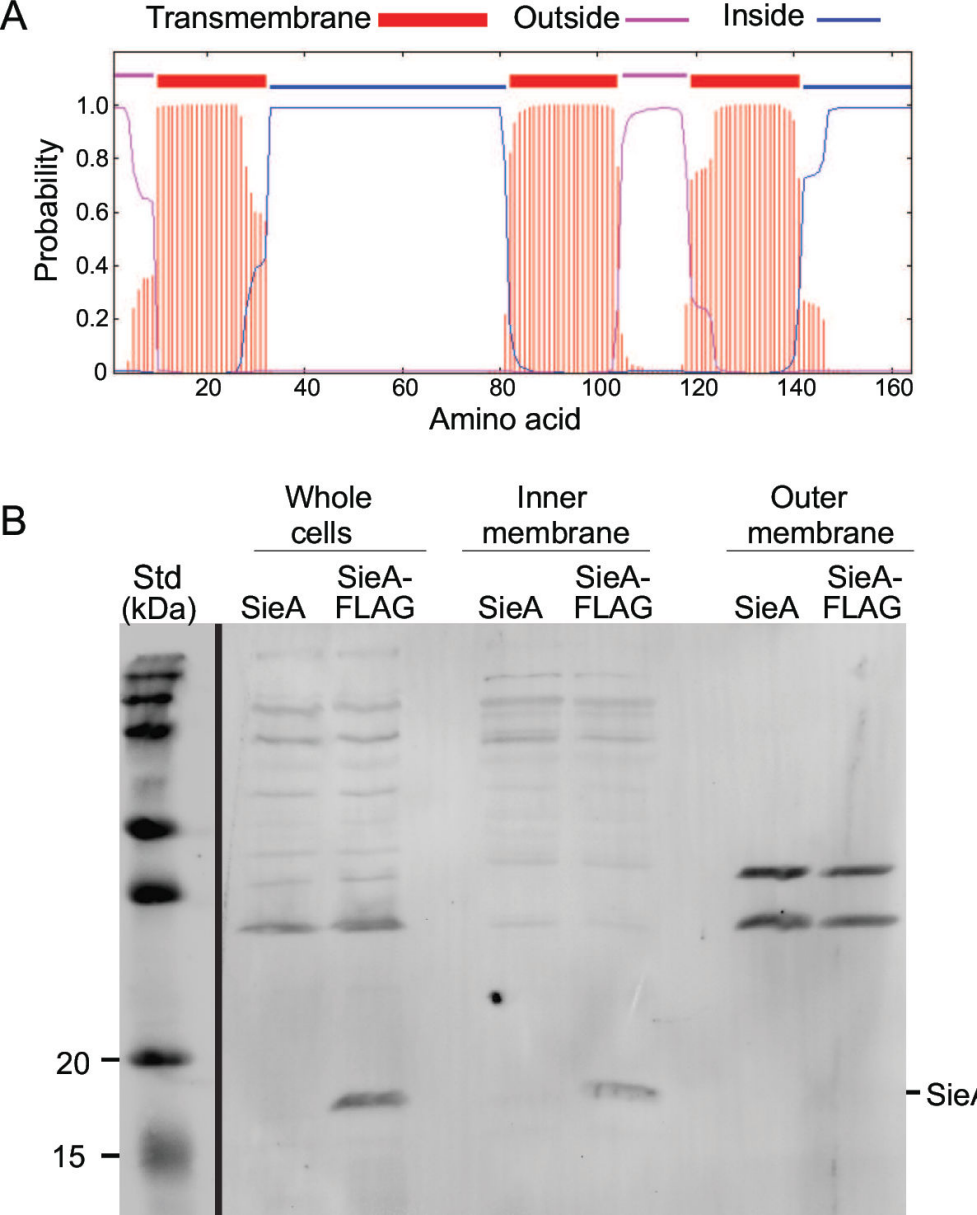
P22 SieA is an inner membrane protein. (A) P22 SieA protein membrane topology. Membrane topology of P22 SieA protein was predicted by a transmembrane hidden Markov model (TMHMM) (61). (B) Cell fractionation. The inner and outer membrane fractions of *S. enterica* strains carrying a P22 *sieA* gene (UB-2520) or a C-terminally FLAG-tagged P22 *sieA* gene (UB-2668) were isolated as described in Materials and Methods. Samples derived from equal numbers of cells of whole-cell lysate and inner membrane and outer membrane fraction proteins were separated by 15% SDS-PAGE. The SDS-PAGE immunoblot was probed with an anti-FLAG monoclonal antibody (Materials and Methods). Precision Plus Protein Kaleidoscope (Bio-Rad) molecular weight markers are indicated on the left. Although some additional bands reacted with the anti-FLAG antibodies, the only difference between the tagged and untagged samples identifies the band that runs at the expected size of SieA protein (about 19 kDa) as the FLAG-tagged SieA protein band.

Although it has no obvious signal sequence, the 162- or 164-amino acid SieA protein (its start codon is uncertain) is predicted to be an integral membrane protein with three transmembrane domains (Fig. 3A). Hofer et al. (36) reported that essentially all SieA proteins are in the *Salmonella* cell membrane pellet fraction under presumably overproducing conditions. That study did not determine whether SieA is in the inner or the outer membrane. In order to sensitively detect the SieA protein, we attached the FLAG epitope-coding sequence (58) to the 3′-terminus of the ectopic chromosomal *sieA* gene (strain UB-2668). This C-terminally tagged SieA has strong superinfection exclusion activity (Fig. 1; Table 2). Inner and outer membrane fractions of isogenic *Salmonella* strains with untagged SieA or FLAG-tagged SieA were prepared according to method 1 of Thein et al. (59) (details in Materials and Methods). The proteins present in the whole cells as well as the inner and outer membrane fractions were displayed by sodium dodecyl sulfate polyacrylamide gel electrophoresis (SDS-PAGE), and the FLAG-tagged SieA protein was identified by immunoblot analysis using monoclonal antibodies directed against the FLAG tag (Fig. 3B). These antibodies produced tagged SieA bands of approximately the same intensity in the whole-cell extract and inner membrane (IM) fraction lanes of the FLAG-tagged strain, but no such band was present in the outer membrane fraction. The strain in which the SieA protein was not tagged showed no immunoblot band, confirming the specificity of the antibodies. Similar experiments with the somewhat less well-documented membrane separation method of Sandrini et al. (60) also indicated that all SieA protein was in the inner membrane (data not shown). We conclude that SieA is an exclusively inner membrane protein.

**TABLE 2:** SieA exclusion insensitive mutants of phage P22

P22 phage genotype	Plating efficiency^*a*^			Phage source^*b*^
	P22 *sieA*^+^ (UB-2520)	P22 *sieA*-FLAG (UB-2668)	*yajC*^–^ (UB-2671)	
“Wild type” (parental P22 UC-0937)	<10^–7^	<10^–7^	<10^–7^	UB-2158
Original escape mutant S1^*c*^	0.04	ND^*d*^	ND	UB-2461
Gene *16* P546S	<10^–5^	<10^–7^	ND	UB-2343
Gene *16* P546S, gene *20* G350D	0.05	0.02	ND	UB-2430
Gene *16* P546S, gene *20* T338I	0.06	0.14	ND	UB-2475
Gene *16* P546S, gene *20* T341I	0.005	0.002	ND	UB-2474
Gene *16* P546S, gene *20* A348T	0.03	<10^–5^	ND	UB-2476
Gene *16* G532W and P546S, gene *20* G350D	0.7	0.3	<10^–7^	UB-2653
Gene *16* G532W, gene *20* G350D	0.1	<10^–5^	ND	UB-2634
Gene *16* G532W and P546S, gene *20* T341I	0.5	ND	<10^–7^	UB-2631

^*a*^ Plating efficiency values in the table are normalized to the number of plaques on strain UB-0002 that has no *sieA* gene. Representative results are shown from three replicate experiments that gave very similar results.

^*b*^ *Salmonella* strains were grown to 2 × 10^8^ cells/mL in 25 mL of Luria-Bertani broth by shaking at 37°C and induced by addition of carbadox to a final concentration of 1.5 mg/mL (62). Shaking was continued at 37°C for 3 h; the cells were lysed by vigorous shaking with 0.5 mL chloroform; cell debris was removed by centrifugation; and the cleared lysate was titered on the indicated strains. The phages are all isogenic derivatives of P22 UC-0937.

^*c*^ Lysogen of original mutagenized tiny plaque phage S1 that carries gene *16* P546S and gene *20* G350D as well as other mutations (see text and Table S1). The seven other original escape phages had plating efficiencies on a *sieA^+^* host that were between 2% and 6% relative to a host with no *sieA* gene.

^*d*^ ND, not determined.

### P22 mutations that allow escape from SieA exclusion

Mutants of phage P22 that escape SieA-mediated superinfection exclusion have been reported but were not characterized (19). Our initial attempts at isolating such mutants by plating P22 *c1*^7^ phages (strain UC-0763 stock grown on *Salmonella* UB-0002) on a *sieA^+^* (UB-2520) lawn generated rare plaque-forming phages at a frequency of less than 1 in 10^8^ infecting phages. Two of these were subjected to whole-genome sequencing and were found to be hybrid phages that contain the P22 early region and the phage Fels-1 virion assembly genes. Fels-1 is a lambda-like prophage that is present in strain UB-0002 (63, 64), and P22-Fels-1 hybrids have been observed previously (63, 65). Unlike those of P22, Fels-1 virions have lambda-like long non-contractile tails (65, 66). The fact that such hybrid phages successfully inject their DNA into cells expressing SieA protein lends additional support to the notion that the P22 virion proteins are the target of SieA exclusion.

Attempts to isolate SieA escape mutants using P22 grown in a prophage-free host strain (UB-2232) did not give rise to any SieA-resistant plaques. A mutagenized stock of clear plaque P22 *c1*^7^ was therefore prepared by growing it on the prophage-free host in the presence of 5-μg/mL nitrosoguanidine. This concentration of mutagen was chosen as it gave the most *amber^+^* of gene *13* in infections by P22 *c1*^7^, *13^–^am*H101 (UC-0011). When these mutagenized P22 *c1*^7^ phage stocks were plated on a *sieA^+^* host, approximately 1 plaque was obtained per 10^8^ applied phages. Eight plaques were picked from two independently mutagenized lysates and purified through three successive single-plaque isolations. These SieA escape mutants made plaques with ≤6% efficiency on a *sieA^+^* relative to isogenic *sieA^–^* host (e.g., see original isolate S1 in Table 2). The eight mutants were subjected to whole-genome sequencing and were found to carry between 7 and 25 new mutations (listed in Table S1). None were sibling plaques as judged from their mutation content. Each of these phages carried mutations in its E-protein encoding genes *16* and *20*, and no other genes were affected in all mutants. All eight mutant isolates have a gp16 P546S substitution, and five also have a gp20 G350D substitution. The other three mutant phages carry one of following gp20 mutations instead of G350D: T338I, T41I, or A348T (Table 2; Table S1). Their universal presence in the mutants suggested that these E-protein changes were likely responsible for the observed partial SieA escape.

In order to prove that the above E-protein changes are responsible for SieA escape, recombineering methods were used to construct isogenic phages that carry the above E-protein mutations and lack the presumably extraneous mutations in the original escape mutant phages (see Materials and Methods). The prophage used for all recombineering in this study was P22 UC-0937. The construction and properties of this phage are described in Fig. S2: it carries the *13^–^amber* mutation that allows control of lysis after lytic growth in hosts that have an *amber* suppressor (above), a *c1* mutation that greatly lowers the frequency of lysogenization and so allows better lytic propagation without affecting the stability of the prophage state, a chloramphenicol resistance cassette that allows positive selection for lysogens, and a *sieA^–^* deletion that ensures robust synthesis of tailspike protein after prophage induction and obviates any potential complications due to SieA protein expression by the phage in the experiments presented below. None of these mutations affects prophage induction or progeny phage production during lytic growth.

The engineered gp16 P546S phage was unable to form plaques on a *sieA^+^* host despite its presence in all eight escape mutants (its absence was confirmed in the starter phage in both of the mutagenized stocks). Each of the four gene *20* mutations was also placed in the prophage in combination with the gene *16* P546S mutation. These four engineered double mutants formed plaques on *sieA^+^* hosts, and we conclude that these amino acid changes in genes *16* and *20* are responsible for the ability to make plaques on *sieA^+^* hosts. Both mutations are required for forming plaques on the *SieA*^+^ host (see below). However, like the original parental escape mutant isolates, plaques were formed only ≤6% as efficiently on a *sieA^+^* host as on a *sieA^–^* host (Table 2).

In order to isolate a P22 phage that forms plaques with higher efficiency on a *sieA^+^* host, the engineered *16* P546S, *20* G350D double-mutant phage (UC-972) was propagated through multiple rounds of infection on a *sieA^+^* host as follows: A 25-mL L broth culture of *Salmonella* UB-2520 was grown to 5 × 10^7^ cells/mL at 37°C and infected with an isolated single plaque of P22 UC-972 from a *sieA^–^* (UB-0002) lawn, and the culture was shaken at 37°C for 5 h. Then, 100 μL of this culture was diluted into a fresh 25-mL culture of the *sieA^+^* UB-2520 and shaken again at 37°C for 5 h. After nine such passages, a plaque was identified whose phage plated with approximately the same efficiency on *sieA^–^* and *sieA^+^* hosts. This phage was subjected to whole-genome sequencing, and it carried only a single new mutation, a substitution that altered gene *16* codon 532 from glycine to tryptophan (strain UC-0979). This triple escape mutant phage makes plaques on a *sieA^+^* host with nearly wild-type efficiency (~70% compared to a *sieA^–^* host), which is approximately 10-fold better than the starting double mutant (Table 2). Phage P22 *16* G532W, *20* G350D produced plaques at only about 10% efficiency on a *sieA^+^* host, indicating that the gp16 G532W change is not sufficient to achieve high-efficiency escape, even in combination with the G350D gp20 change. A triple escape mutant with a different gp20 mutation, P22 *16* G532W, P546S, and *20* T341I (phage UC-0980), made plaques on a *sieA^+^* host with essentially full efficiency (Table 2), indicating no allele specificity in these gene *20* mutation combinations. Since amino acid changes in E-proteins gp16 and gp20 confer escape from SieA exclusion, we conclude that SieA very likely blocks P22 DNA injection by interfering with the functions of these two proteins and that the mutations avoid this interference. The specific roles of gp16 and gp20 are discussed below.

However, we found that the P22 *16* G532W, *16* P546S, *20* G350D SieA escape phages remain partially defective for K^+^ release (Fig. 2A). The triple-mutant phage released K^+^ markedly slower than the *16^+^*, *20^+^* phage, even in the absence of the host *sieA* gene. At MOI = 5, release was slower and less extensive than release by the parental *16^+^*, *20^+^* phage on host with or without SieA. At a higher MOI of 10, the rate of K^+^ release by the triple mutant increased about fourfold on the *sieA^+^* host, and the extent of release nearly reached the extent of “wild-type” phage on *sieA^–^* host after 30 min (Fig. 2A). Thus, although the P22 triple mutant makes plaques with nearly wild-type efficiency, it is partially impaired for K^+^ release and thus probably has somewhat impaired (slower?) DNA injection even on a *sieA^–^* host.

Bohm et al. (67) found the host inner membrane protein YajC to be essential for P22 DNA injection. It is thus a candidate for an inner membrane feature that the P22 injection apparatus (see below) contacts. However, neither of the above P22 SieA-resistant triple-mutant phages made plaques on a *yajC^–^* host (Table 2), and like wild-type phages [see reference (46)], they did not release potassium ions from *S. enterica* whose *yajC* gene had been deleted (data not shown). Thus, these SieA escape mutations do not bypass the YajC requirement for infection.

### SieA target and host specificity

#### P22 SieA is specific for P22-like phages

Infection by P22-like short-tailed *Salmonella* phages L, MG40, and MG178 is blocked by the *sieA^+^* gene expressed from a P22 prophage (19). We confirmed that these three phages as well as P22-like *Salmonella* phage LP7 (48) are unable to form plaques on *Salmonella* with an ectopically expressed *sieA* gene (UB-2520). On the other hand, we found that the following non-P22-like tailed phages make plaques with the same efficiency on *sieA^–^* (UB-0002) and *sieA^+^* (UB-2520) hosts: *Myoviridae* phages Det7 (45) and Felix-O1 (46), *Siphoviridae* phages ES18 (68) and 9NA (44), and *Podoviridae* T7-like phage SP6 (52). Susskind et al. (19) previously reported that Felix-O1 was not affected by SieA expressed from a prophage. Since SP6 is not affected, the P22 SieA protein does not exclude all short-tailed phages and appears to be specific for P22-like phages.

### P22 SieA is functional in *E. coli*

In order to test the ability of *Salmonella* phage P22 SieA to exclude P22-like phages in another bacterial host species, *E. coli*, we inserted the P22 *sieA* gene into the chromosomes of the hosts of *E. coli* P22-like phages CUS-3 (type K1 strain EV36 43]) and HK620 (type H strain 2158 [41]). These two phages have tailspikes that bind different polysaccharides and therefore cannot infect each other’s host or *Salmonella*. CUS-3 and HK620 plate with efficiencies of ≤10^–8^ and about 0.3, respectively, on their *sieA^+^* host relative to the *sieA*^−^ host (Table 3). Thus, SieA from *Salmonella* phage P22 can function in *E. coli* since it excludes CUS-3 quite efficiently, but it did not function well against HK620 infection in its *E. coli* host. It was not clear from this result whether the failure to exclude HK620 was due to this phage’s insensitivity to P22 SieA exclusion or the failure of the *sieA* gene to be expressed or to function in this host, but the hybrid phage experiments below strongly suggest that exclusion failure is responsible.

**TABLE 3:** P22 SieA protein functions in *E. coli*

*S. enterica* host	No *sieA*^*a*^	P22 *sieA*^+^
	(UB-0002)	(UB-2520)
P22 “WT” (UC-0937)	1	<10^–8^
*E. coli* 2158 host	No *sieA*	P22 *sieA*^+^
	(UB-1702)	(UB-2478)
HK620 WT	1	0.33
*E. coli* EV36 host	No *sieA*	P22 *sieA*^+^
	(UB-1957)	(UB-2558)
CUS-3 WT	1	<10^–8^

^*a*^ Values in the table are plating efficiencies relative to the *sieA^–^* strain. Representative results are shown from three replicate experiments that gave very similar results.

### Hybrid P22 phages that carry foreign E-protein genes

**Fig 4:**
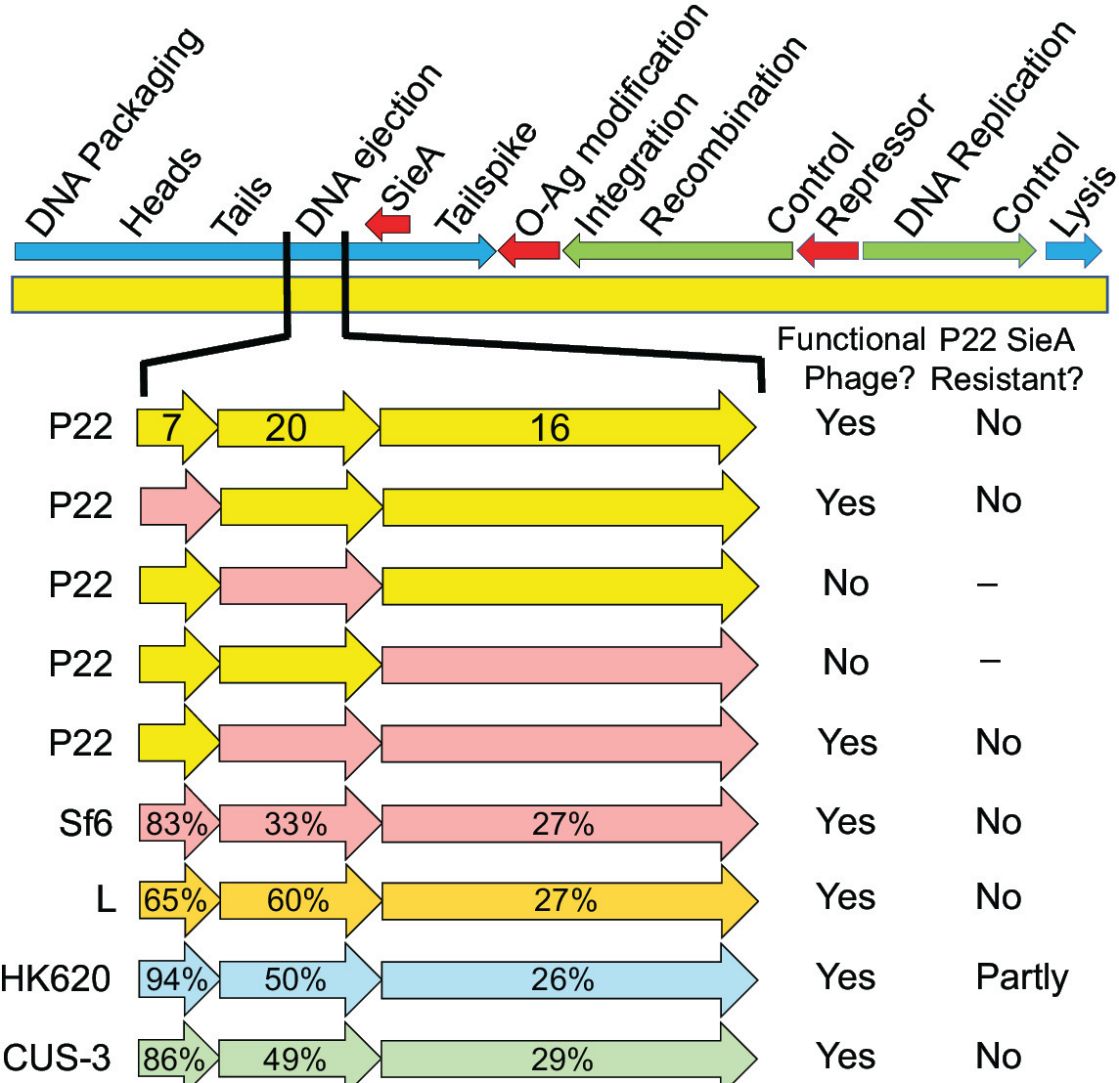
Phage P22 with foreign ejection protein genes. The phage P22 genome is diagrammed above with the location of the *sieA* genes indicated. Red arrows indicate regions expressed from the prophage, while green and blue arrows are regions expressed during early and late lytic growth, respectively. Below, replacement E-protein genes from phages Sf6 (strains UB-2349, UB-2416, UB-2417, UB-2418, and UB-2669), L (UB-2464), HK620 (UB-2537), or CUS-3 (UB-2614) are shown in different colors. P22 gene names are indicated on the P22 genes, and percent identities to their P22 homologs are indicated on the foreign genes. The viability and SieA resistance phenotypes are summarized on the right (see also Table 4).

To further examine SieA target specificity and the idea that SieA inhibits E-protein function, we constructed and analyzed hybrid P22 phages whose E-protein genes were replaced by rather distant homologs from P22-like phages. Recombineering methods were used to construct hybrid P22 prophages in which all three E-protein genes (*7*, *16*, and *20*) were replaced by the parallel E-protein genes from *Shigella* phage Sf6 (51), *Salmonella* phage L (69), or *E. coli* phages CUS-3 (43) and HK620 (41). These four hybrid prophages all produced essentially normal yields of functional phages upon induction with mitomycin C (Table 4; Fig. 4). As expected from their ability to form plaques, SDS-PAGE analysis showed that the foreign E-proteins were in fact incorporated into the hybrid virions (we note that Sf6 gp20 co-migrates with P22 coat protein in SDS-PAGE, so its presence could not be determined; data not shown).

**TABLE 4:** Functionality of P22 ejection protein hybrid phages

Ejection proteins^*a*^	Titer (PFU/mL)^*b*^				Phage source^*b*^
	No sieA (UB-0002)	P22 sieA+ (UB-2520)	P22 sieA + FLAG (UB-2668)	HS1 sieA+ (UB-2621)	
P22 gp7, gp20 and gp16	5 × 10^10^	<10^4^	<10^4^	<10^4^	UB-2158
P22 gp20 and gp16, Sf6 gp7	1 × 10^11^	<10^4^	<10^4^	<10^4^	UB-2416
P22 gp7 and gp20, Sf6 gp16^*c*^	<10^4^	ND^*d*^	ND	ND	UB-2418
P22 gp7 and gp16, Sf6 gp20^*c*^	<10^4^	ND	ND	ND	UB-2417
P22 gp7, Sf6 gp16, and gp20	5 × 10^10^	<10^4^	<10^4^	<10^4^	UB-2669
Sf6 gp7, gp20, and gp16	2 × 10^9^	<10^4^	<10^4^	<10^4^	UB-2349
L gp7, gp20, and gp16	1 × 10^10^	<10^4^	<10^4^	<10^4^	UB-2464
CUS-3 gp7, gp20, and gp16	1 × 10^11^	<10^4^	<10^4^	<10^4^	UB-2614
HK620 gp7, gp20, and gp16	2 × 10^10^	3 × 10^9^	4 × 10^9^	2 × 10^9*e*^	UB-2537

^*a*^ E-proteins encoded by the prophages in the strains in the rightmost column.

^*b*^ The lysogens indicated in the rightmost column were grown and induced to lytic growth as described in Table 2. Lysates were titered on the host strains indicated above the columns. Representative results are shown from three replicate experiments that gave very similar results. These phages are isogenic with P22 UC-0937 (see text).

^*c*^ The prophages in UB-2417 and UB-2418 do not produce functional virions upon induction but produce similar numbers of non-infectious virion-like particles.

^*d*^ ND, not determined.

^*e*^ Tiny plaques.

Infection of *Salmonella* by three of the four above hybrid phages was efficiently blocked by P22 SieA; however, the HK620 hybrid made plaques with about 10% efficiency on stains with SieA relative to strains without SieA (Table 4). This observation is consistent with HK620’s similarly reduced plating efficiency on its *E. coli* host when the *sieA* gene was present (above). The finding that P22 virions with HK620 E-proteins are relatively weakly sensitive to P22 SieA exclusion further supports the idea that SieA protein interacts with E-proteins during DNA delivery into cells.

We also note that when the individual P22 E-protein genes were replaced by their Sf6 homologs, only the prophage with the Sf6 gene *7* homolog gave a normal progeny yield, while induction of prophages with only Sf6 gene *16* or gene *20* did not produce plaque-forming phages. The non-functional virions produced after induction of the P22 prophage with only Sf6 gene *16* contained Sf6 gp16 by SDS-PAGE analysis (data not shown); thus, their defect was not due to the inability of Sf6 gp16 to assemble into the particles but rather due to its inability to function after assembly (but as mentioned above, Sf6 gp20 could not be measured in phages that carry only Sf6 gene *20*). However, a prophage with Sf6 genes *16* and *20* (UB-2669) produced functional progeny (Table 4; Fig. 4). The simplest explanation for this observation is that gp16 and gp20 assemble into virions independently but must interact in the virion and/or during DNA delivery, and P22 gp16 and gp20 cannot interact with Sf6 gp20 and gp16, respectively. Such a functional interaction helps to explain why mutations in genes *16 and 20* overcome SieA exclusion.

### Diversity of *sieA* genes

**Fig 5:**
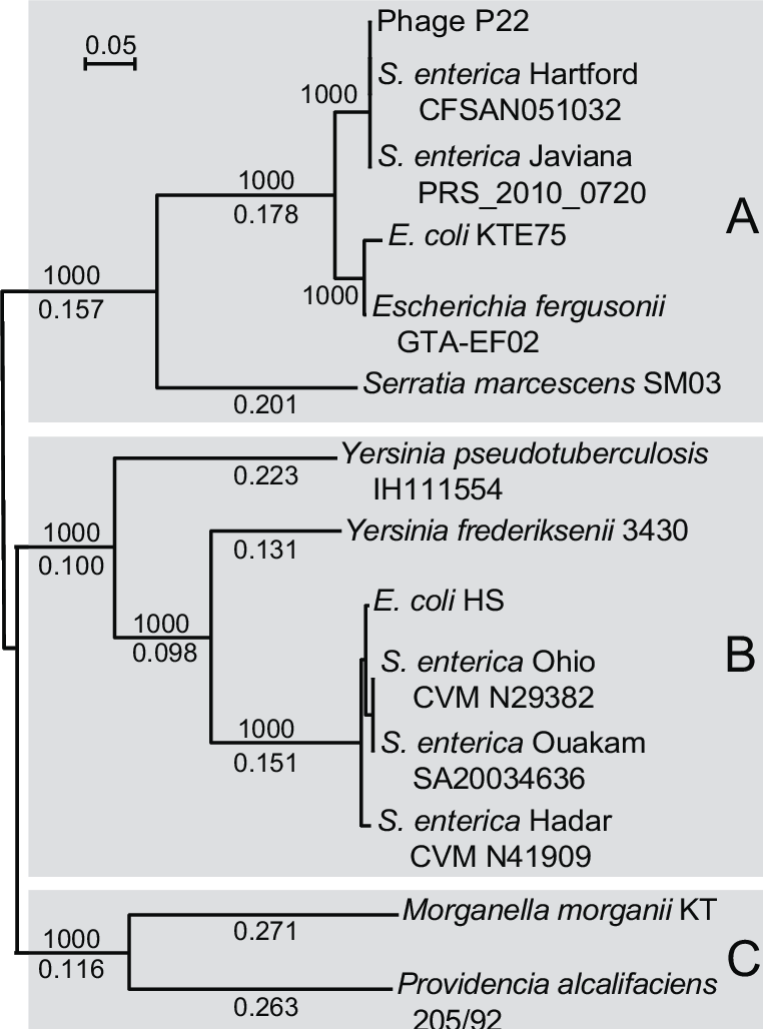
Neighbor-joining tree of representative SieA proteins. An unrooted Clustal neighbor-joining tree (71) of SieA proteins chosen to display the extent of their diversity is shown. The tree shows selected bootstrap values out of 1,000 trials above the branches and fractional distances below the branches. Gray boxes indicate the three sequence classes of SieA protein homologs. A bar representing a fractional distance of 0.05 is shown in the upper left. Table S2 gives locus_tags for the SieA sequences.

Not all P22-like phages carry a *sieA* homolog. A search of the current public sequence database with BLASTp (70) found that 22 of the 66 P22-like phages whose genomes have been reported carry *sieA* genes (as of 7 June 2023). These 22 phages all infect *Salmonella*, and their SieA proteins are close relatives of P22 SieA. Nonetheless, more diverse *sieA*-like genes are present in bacterial genomes in the current public database. Most such *sieA*-like genes are present in *S. enterica* and *E. coli* genomes, but a few are in seven other bacterial genera: *Shigella*, *Serratia*, *Yersinia*, *Pectobacterium*, *Providencia*, *Proteus*, and *Morganella* (all members of the family Enterobacteriacae). In all cases that were examined in detail, including over 20 each of th*e Salmonella* and *Escherichia sieA*-like genes and 18 of those in the last 7 genera, the *sieA* gene resides in a P22-like prophage and occupies a position similar to that of P22, the *sieA* gene between the homologs of P22 genes *16* and *9*. There is substantial variation among these prophage SieA-like proteins, and they are present as three main types—types A, B, and C—that are only about 25% identical to one another in amino acid sequence (Fig. 5). The *S. enterica* and *E. coli* prophage SieA proteins are either type A or B, and type C is currently limited to the Providencia, Morganella and Proteus genera.

The three SieA types have very similar transmembrane helix predictions by TMHMM analysis (61) (Fig. S3); however, types B and C have about 30- and 40-amino acid N-terminal extensions, respectively, relative to type A (P22 SieA is type A), and type C also has about 10 additional amino acids at the C-terminus. The extra N-terminal amino acids in types B and C contain an additional predicted transmembrane section and a putative N-terminal cytoplasmic region. Comparison of the three types shows weak sequence similarity scattered across the proteins with a somewhat more highly conserved motif, FxRYPxEAxxFNK, between P22 SieA residues 145–157 that is predicted to be on the cytoplasmic side of the membrane.

A type B SieA protein is encoded by the P22-like prophage HS1 in the *E. coli* strain HS genome (57, 72, 73). It is only 24% identical to P22 SieA protein. We examined the function of the HS1 *sieA* gene in more detail by polymerase chain reaction (PCR) amplifying its DNA along with 106 bp of upstream sequence from strain HS (UB-1732, locus_tag EcHS_A0321 in accession no. CP000802) and inserting it into the same *galK* location in the *S. enterica* chromosome as the P22 *sieA* gene described above. *Salmonella* phage P22 (Table 4, top line) and P22-like phages L, MG40, MG178, and LP7 (not shown) were all fully excluded by HS1 SieA. In addition, like P22 SieA, HS1 SieA blocks infection of *S. enterica* by P22 hybrid phages that carry all three L, Sf6, and CUS-3 E-protein genes but again did not completely block the HK620 hybrid which plated with about 10% efficiency (Table 4). Despite their substantial amino acid sequence differences, the P22 and HS1 SieA proteins bestowed indistinguishable exclusion phenotypes in these experiments.

## DISCUSSION

### Inner membrane protein SieA blocks P22 DNA injection

We have shown that phage P22-encoded superinfection exclusion protein SieA is an inner membrane protein that specifically blocks injection of DNA into the cytoplasm of *S. enter*ica and *E. coli* cells from the virions of P22-like phages, and that no other phage-encoded proteins are required for this exclusion. We isolated P22 SieA escape mutants and hybrid phages in which changes in gp16 and gp20 overcome or partially overcome the SieA block. Three P22 E-proteins, gp7, gp16, and gp20, are present in P22 virions and are released from the virion after adsorption but before DNA injection (27, 32, 39, 74). Upon release from the virion, the E-proteins assemble into a hollow conduit for DNA passage from the virion through the host cell membranes, peptidoglycan and periplasm, into the cytoplasm (25–27). The observations here that (i) P22 infection by virions lacking E-proteins mimics the SieA block phenotype, (ii) P22 hybrids with phage HK620 E-proteins are partially resistant to SieA, and (iii) mutations that alter P22 gp16 and gp20 confer SieA resistance strongly suggest that SieA protein interferes with assembly of the E-protein conduit or with conduit function after its assembly.

Our observation that P22 SieA is an inner membrane protein is a first for a short-tailed phage exclusion protein, but similar exclusion mechanisms may also affect long non-contractile tailed phages. *Lactococcus* phage Tuc2009 and *Streptococcus* phage TP–J34 also encode superinfection exclusion membrane proteins that block DNA injection into their Gram-positive hosts (9, 10, 75, 76), and phage HK97 superinfection exclusion protein gp15 is also predicted to be an inner membrane protein in its Gram-negative *E. coli* host (8). These three phages all have long non-contractile tails, and their superinfection exclusion proteins appear to be specific for other long-tailed phages. It has been proposed that the long non-contractile tail tape measure proteins might form a transperiplasm DNA injection conduit for this type of phage (9, 77–79). These superinfection exclusion blocks can be overcome by alterations in the TP–J34 tape measure protein (9), and HK97 hybrids that have the phage HK022 tail tube and tape measure genes escape gp15 exclusion (8). Similarly, although it is very different from P22, the short-tailed phage T7 also builds a conduit whose atomic structure has been determined (29, 30, 80). Its component proteins (T7 gp14, gp15, and gp16) have little recognizable sequence similarity to the P22 E-proteins. Thus, even though their component proteins have few obvious similarities in the above different phage types, the assembly of a transperiplasm DNA injection conduit seems likely to be a feature shared by all phages with short and long non-contractile tails.

### Roles of P22 ejection proteins and SieA-mediated exclusion

#### Gene *7* protein

There is no evidence that gp7 is directly involved in SieA exclusion. The relatively low extant diversity of gp7 suggests that it may interact with something more evolutionarily conserved than the host cell envelope, and an excellent candidate is the unusually highly conserved gp10 in the P22 virion’s tail (72). Indeed, gp7 may form the short-tail extension from the gp10 tail core seen in P22 virions that have spontaneously lost their DNA (81) and the phage virion proximal portion of the conduit (28).

#### Gene *20* protein

Purified phage Sf6 gp20 assembles *in vitro* into a 15-nm-long tube that has an inner diameter of about 2.5 nm (82). Even though this may not be long enough to reach across the entire periplasmic space, which in *E. coli* is 18–25 nm (80, 83, 84), this structure probably forms at least part of the transperiplasm DNA delivery conduit, since the putative conduit is missing in P22 *20^–^* mutants (28). The gp20 tube is unlikely to be assembled with stacked globular gp20 units; its variable diameter fits much better with a model in which each gp20 polypeptide extends from one end of the tube to the other. This model also allows a facile explanation of how gp20 can tolerate the apparently random exchange of many “very different intragenic” mosaic modules (72) as follows: in a homo-multimeric structure built from parallel extended proteins that reach from one end of the structure to the other, most interactions will be short-range intra-subunit interactions between amino acids close to one another in the sequence and inter-subunit contacts between laterally aligned adjacent neighbors. Thus, any section of the protein could successfully be replaced by another sequence, even a very different sequence, as long as the new segment has the ability to maintain the side-by-side, self-self interactions. If the gp20 tube is built from extended proteins, then the mutations in gp20 at codons 338, 341, 348, and 350 that help overcome the SieA block would be rather near an end of the gp20 tube (P22 gp20 is 471 amino acids long). We note that the C-terminal region of gp20 is essential for its function, since removal of the C-terminal 33 or 66 amino acids of P22 gp20 by nonsense mutations *am*H1032 and *am*L100, respectively, results in non-functional virions (mutant sequences determined in this study).

#### Gene *16* protein

Thomas and Prevelige (85) reported that purified gp16 forms a rod-like assembly, but its structure has not been further investigated. Wang et al. (28) reported that the transperiplasmic conduit is at least mostly formed in the absence gp16 and suggested that it resides in the inner membrane where the DNA would enter the cytoplasm. In agreement with this idea, Perez et al. (86) showed that gp16 associates *in vitro* with *Salmonella* membrane preparations and that gp16 facilitates *in vitro* DNA transport into liposome vesicles under certain conditions. In addition, a P22 mutation (tdx-1) that may affect circularization of generalized transducing DNA was reported to affect gp16 (87), which is consistent with an alteration in DNA handling during or immediately after entry. The gp16 SieA-resistant mutations G532W and P546S probably do not affect the gp16 membrane association since the protein truncation experiments of Perez et al. (86) found that C-terminal amino acids 476–609 are not required for gp16 membrane association *in vitro*.

### SieA and P22-like phage E-protein variation

We previously found that genes *16* and *20* are the among the most genetically variable parts of the P22-like virion assembly gene cluster; only the tailspike O-antigen receptor-binding domain is more diverse (72). It is perhaps surprising that very different E-proteins can successfully substitute for the P22 E-proteins in virion assembly and DNA injection (above). The E-proteins of the five phages examined here—P22, L, Sf6, HK620, and CUS-3—are quite divergent and highly mosaically related (72). The most highly conserved of these E-proteins are the gp7 homologs, which range from 65% to 94% identical to one another in these five phages; it is not surprising that Sf6 gp7 can successfully substitute for the 83% identical P22 gp7 (Fig. 4). The more diverse gp16 proteins are only 22%–26% identical to one another, except for L and Sf6 gp16’s, which are 75% identical. The gp20 proteins are 21%–60% identical to one another, except for the CUS-3 and HK620 proteins, which are 98% identical. Since the CUS-3 and HK620 gp7 proteins and gp20 proteins are 90% and 98% identical, respectively, the ability of the HK620 E-proteins to mediate partial escape from SieA exclusion, while CUS-3 E-proteins do not, likely resides in their gp16 proteins, which are only 26% identical. In spite of these differences, among the gp16 and gp20 amino acids substituted in the SieA escape mutants, P22 gp16 G532 is conserved in all five of the above phages and P22 gp20 T338, T341, and G350 are conserved in three [by Clustal alignment (71)], suggesting that those SieA target residues may have conserved functions.

We showed that the type A SieA encoded by phage P22 and the type B SieA encoded by prophage HS1, which are only 24% identical, have indistinguishable effects on P22 and the four P22 hybrid phages with different E-proteins. The interactions that mediate SieA exclusion must be quite robust since (i) three amino acid changes in the P22 E-proteins are required to fully overcome the plaque-forming block, and (ii) even with all three E-protein amino acid changes (above), the K^+^ release is significantly slowed by the presence of SieA. In addition, the very different P22 and HS1 SieA proteins can both successfully exclude phages with divergent gp16 and gp20 proteins. If SieA interacts with the E-proteins as our results suggest, then it is difficult to understand how such interactions are maintained in the face of such extensive E-protein and SieA diversity. Understanding the details of these interactions will require further investigation.

### Mechanism of SieA exclusion at the inner membrane

The presence of SieA protein in the inner membrane suggests that it interferes with DNA injection at that location. High MOI partially overcomes SieA exclusion of both infecting phage and generalized transducing particles (18, 23, 24), suggesting that high levels of infecting E-proteins are able to saturate a limited number of inhibitory SieA protein molecules. This notion is consistent with transcriptomics data that show that *sieA* is not highly transcribed in the lysogen (88), which in turn lends further credence to the idea that SieA interacts directly with the P22 gp16 and gp20 transperiplasm conduit proteins.

An alternative hypothesis is that SieA occludes a required interaction between another host inner membrane component such as YajC and the distal end of the DNA delivery conduit. If YajC concentrations are in excess of SieA, which is consistent with transcriptomics data, then at high MOI, there would be a higher probability that an infecting phage DNA conduit can “find” a rare unblocked YajC. Interestingly, *sieA* is also upregulated during the course of prophage induction (88), suggesting that its exclusionary role becomes even more critical later in infection.

## MATERIALS AND METHODS

### Bacteria and phage strains

Bacteria and phage strains and their sources are listed in Table 1. S. *enterica* LT2 *supE* strain UB-0002 was used to propagate phage P22 strains, and *E. coli* UB-0049 was used to carry plasmids and as transformation recipient during plasmid construction. All plasmid and phage constructs were confirmed by determination of the sequence of the modified region after PCR amplification or by whole-genome Illumina sequencing at the University of Utah Sequencing Core Facility.

### Strain construction by recombineering

The *galK* recombineering methods used to modify phage P22 prophages were described in Padilla-Meier et al. (89) and Leavitt et al. (90). Briefly, in a *Salmonella* strain that carries the desired prophage (P22 UC-0937 here) and in which the native *galK* gene has been replaced by a TetRA tetracycline resistance cassette (91), the modification target was first replaced by a functional *galK* expression cassette (92) amplified by PCR using primers that have ≥40-bp 3′-tail sequences with target homologies that program its precise insertion into the desired location by homologous recombination. A DNA of choice was then used to recombinationally replace the *galK* cassette by selecting for the concomitant loss of the *galK* gene by growth in the presence of 2-deoxygalactose (92). Plasmid pKD46 (93) was present to increase the efficiency of recombinational replacements but was removed before any prophage induction experiments. Replacement DNAs for constructing P22 prophages with specific point mutations were created by using a synthetic double-stranded oligonucleotide containing the desired modification and ≥80-bp identity with the target. Replacement DNAs for constructing P22 prophages with E-protein genes from other phages were PCR amplified from DNA of the other phages with appropriate 3′-tails. The genome structures of these hybrid phages were confirmed by Illumina whole-genome sequencing. In the Sf6 hybrid phages that did not give plaques (UB-2417 and -2418), the replacements, including both boundaries, were confirmed by PCR amplification and sequencing by Sanger et al. (94) methods. In all cases, except the phage L construct (UB-2464), the gene replacements were precise. In the latter case, the recombination that inserted the L E-protein genes included the first 23 codons of tailspike-encoding L gene *9*, which is downstream of gene *16*, so this hybrid prophage contains the *7*, *20*, and *16* genes of phage L, the 836 bp between its gene *16* and gene *9*, as well as this part of gene *9* (the first 23 amino acids of P22 and L tailspikes are identical, but there are four synonymous differences in this region).

To create ectopic *sieA* gene-carrying *S. enterica* and *E. coli* strains, the bacteria were converted to *galK^–^* by recombineering replacement of the native *galK* gene by an appropriately PCR-amplified P22 *sieA* gene (with or without C-terminal FLAG tag amino acid codons in the 3′-tail of the relevant primer) or HS1 *sieA* gene (95) followed by selection with 2-deoxyglactose.

### Nucleotide sequencing and sequence analysis

Sanger sequencing (94) of amplified PCR product DNAs and whole-genome Illumina sequencing were performed by the High Throughput Genomics Core Facility, University of Utah. The latter utilized MiSeq 150-bp paired-end methodology with a 350-bp insert library. Quality controlled, trimmed reads were assembled into a single, circular phage sequence contig with >400-fold coverage using Geneious (v.9.0.5) (96).

SieA protein homolog identification was carried out by examining the results of BLASTp (70) searches of the extant sequence database through the National Center for Biotechnology Information (NCBI) website (https://blast.ncbi.nlm.nih.gov/Blast.cgi), and the neighbor joining tree was constructed with Clustal (71). Membrane topology of SieA protein was predicted by TMHMM (61) at the online site http://www.cbs.dtu.dk/services/TMHMM/.

### Cell fractionation, protein electrophoresis, and immunoblot analysis

The IM and outer membrane (OM) fractions of strains UB-2520 and UB-2668 were separated according to method one described by Thein et al. (59). Triton X-100 was removed from the IM-containing supernatant with a Pierce detergent removal spin column (Thermo Fisher Scientific). The OM pellets were resuspended in a small volume of sterile distilled water by gentle shaking overnight at 4°C, applied to the top of 35%–40% OptiPrep (Sigma) density gradients prepared in 30 mM MgCl_2_, 120 mM Tris-HCl, pH 8, and spun in a Sorvall MX120 ultracentrifuge at 4°C for 1 h at 100,000 rpm. The gradients were fractionated, and the resulting fractions were analyzed by SDS-PAGE after boiling for 3 min at 90°C in 3× sample buffer. The IM- and OM-containing fraction was stored at −20°C until Western blot analysis.

For Western blot analysis, equal amounts of the whole-cell lysates as well as the IM- and OM-containing fractions of strains UB-2520 and UB-2668 were separated on a 15% acrylamide SDS-PAGE gel. Transfer to an Immobilon-PSQ membrane (Millipore) was carried out using a TE22 transphor electrophoresis unit (Hoefer Scientific) in 150 mM glycine, 20 mM Tris base, pH 8.3, 20% methanol, 0.01% SDS overnight at 30 V at constant 0.1 amp with stirring. The membrane was blocked with 10% non-fat milk in 20 mM Tris, pH 7.6, 150 mM NaCl for 1 h with gentle shaking. The blot was washed for 5 min twice with Tris buffered saline with Tween 20 (TBS/T) (20 mM Tris-HCl, 150 mM NaCl, pH 7.6, plus 0.01% Tween 20). The FLAG-tag monoclonal antibody conjugated to DyLight 800 4X PEG (Thermo Fisher Scientific) was diluted to a final working concentration of 1:1,000 in 1% non-fat milk in TBS/T. The membrane was incubated in the antibody solution for 2 h at room temperature in the dark with gentle agitation and then washed for 5 min three times in TBS/T. The blot was rinsed and stored in TBS and visualized using a ChemiDoc MP imaging system (Bio-Rad).

### Analysis of transcriptomics data

Raw RNA-seq reads pertaining to wild-type P22 infection and prophage induction (88) were obtained from the NCBI SRA database (BioProject accession no. PRJNA737196). Raw reads were processed using BBDuk (v.38.84) in Geneious Prime (v.2022.2.2) to remove adapter sequences and low-quality reads. Individual processed read sets from either the infection or induction conditions were mapped to the *Salmonella enterica* subsp. *enterica* serovar Typhimurium strain LT2 (RefSeq accession no. NC_003197.2) and *Salmonella* phage P22 genomes (GenBank accession no. BK000583.1) using Geneious Mapper with default settings. RNA expression levels were obtained using the “calculate expression values” function in Geneious with default settings, and differentially expressed genes were identified using the R package DEseq2 (97) within Geneious.

## SUPPLEMENTAL MATERIAL

Supplemental material mbio.02169-23-s0001.docx Supplemental tables and figures.

ASM does not own the copyrights to Supplemental Material that may be linked to, or accessed through, an article. The authors have granted ASM a non-exclusive, world-wide license to publish the Supplemental Material files. Please contact the corresponding author directly for reuse.
