# Supplementary material for: Bacteriophage P22 SieA-mediated superinfection exclusion
Source: mBio. 2024 Jan 18;15(2):e02169-23. doi: 10.1128/mbio.02169-23 (PMC10883804; doi:10.1128/mbio.02169-23)
Supplement: Supplemental material — Supplemental tables and figures. [file mbio.02169-23-s0001.docx]

**These Supplementary Materials contain the following:**

Page 2 Table S1 Changes in original mutants of phage P22 *c1*^7^ that overcome the SieA block

Page 3 TableS2 Prophage *sieA* gene locus_tags

Page 5 Figure S1 The P22 SieA protein has a minimal effect on *S. enterica* growth

Page 8 Figure S2 Construction of phage P22 phage UC-0937 - an improved P22 prophage recombineering substrate

Page 9 Figure S3 Predicted membrane topology of the three SieA sequence types

Page 10 References

**Table S1**

**Changes in original mutants of phage P22 c1^7^ that overcome the SieA block**

gp20 gp16

Thr338Ile Thr341Ile Ala348Thr Gly350Asp Pro546Ser Number of other changes

Mutant

S1 – – – + + 18

S2 – + – – + 23, including 1 gene *20* synonymous

S5 – – – + + 5, including 1 gene *16* Gly191Ser

SA – – – + + 9, including 2 gene *16* synonymous

SB – – – + + 6

SD + – – – + 9, including 1 gene *16* synonymous

SE – – – + + 11 including 1 gene *16* synonymous

SF – – + – + 14 including gene *7* Ala105Thr and 1 gene *16* synonymous

**Table S2**

**Prophage *sieA* gene locus_tags**

Bacterial Isolate *sieA* homologue locus tag

*E. coli* HS EcHS_A0321

*E. coli* KTE75 A1UM_02908

*Escherichia fergusonii* GTA-EF02 VP22_0209700

*Morganella morganii* KT MU9_2893

*Providencia alcalifaciens* 205/92 HMPREF1563_1315

*S. enterica* Hadar CVM N41909 DE81_11120

*S. enterica* Hartford CFSAN051032 BUJ28_004415

*S. enterica* Javiana PRS_2010_0720 SEEJ0720_04091

*S. enterica* Ohio CVM N29382 DD58_22310

*S. enterica* Ouakam SA20034636 LFZ36_17085

*Serratia marcescens* SM03 A8A12_01925

*Yersinia frederiksenii* 3430 ERS008545_03773

*Yersinia pseudotuberculosis* IH111554 ERS008459_01168

**Figure S1. The P22 SieA protein has a minimal effect on *S. enterica* growth.**

Absorbance of bacterial cultures was followed with a Bioscreen C automated growth analysis system (Growth Curves USA, Piscataway, NJ) at 37ºC in rich medium (L broth). *Salmonella* LT2 *galK^–^* strain UB-2666 and the isogenic *sieA^+^* UB-2520 have very similar growth curves. UB-2665, a restriction minus version of UB-2666 that is also missing the Fels2 prophage, shown for comparison, has a similar growth curve.

Hofer *et al.* (1995) reported that expression of SieA from a *tac* promoter on a multicopy plasmid caused a growth rate reduction but did not quantify the effect. The figure shows that the presence of the *sieA* gene in its chromosome has no strong effect on *S. enterica* cell growth at 37ºC in rich medium. Thus, although it may be more detrimental in high levels, a single *sieA* gene has little effect on *Salmonella* growth under these conditions.

**Figure S2. Construction of phage P22 phage UC-0937 - an improved P22 prophage recombineering substrate.**

We previously described hybrid phage constructs in parental prophage P22 *13^–^am*H101, *15*^–^∆sc302::KanR, *sieA^–^∆*1 (P22 strain UC-0911 prophage in bacterial strain UB-1790; Padilla-Meier *et al.*, 2012; Leavitt *et al.*, 2013a) in which the *13^–^amber* mutation allows control of lysis after lytic growth, the kanamycin resistance cassette allows positive selection for lysogens, and the *sieA^–^* deletion ensures robust synthesis of tailspike protein after prophage induction (see Adams *et al.*, 1985). In order to avoid the use of citrate plates for titering P22 phages (made necessary by the *15*^–^ deletion in phage UC-0911) and to allow more efficient lytic propagation of functional engineered phages, the genetic modification experiments here were performed with a modified prophage that also carries the *c1*^7^ clear plaque mutation and a chloramphenicol resistance cassette (orf25::CamR-EG1) that does not disrupt P22 gene *15* replaces the KanR cassette. The only P22 open reading frames replaced by the CamR cassette are orf25, orf80 and *pid*; the former two are not required for lysogeny or lytic growth but have not been studied further (Casjens *et al.*, 1989; Eppler *et al.*, 1991), and the latter is a nonessential gene that derepresses the host’s *dgo* operon in P22 pseudolysogen cells (Cenens *et al.*, 2013). The C1 protein stimulates establishment of the prophage state but is not required for its maintenance (Susskind and Botstein, 1978). This P22 *c1*^7^, *sieA^–^∆*1, *13^–^am*H101, orf25::CamR-EG1 phage was named P22 UC-0937; it is present as the prophage in *Salmonella* strain UB-2158. Its construction is diagrammed in figure S2, and the details of its construction and characterization are described in the following paragraphs.

In figure S2 genomes are not drawn to scale, and the pink TetRA insertion indicates the tetracycline resistance cassette that replaces (and thus inactivates) the host *Salmonella* *galK* gene. The chloramphenicol resistance (CamR) cassette of *Salmonella* strain UB-1760 was PCR amplified using primers with appropriate 3'-tail sequences, and this DNA was used to electroporate a P22 *13^–^am*H101 (phage UC-0033) lysogen, and chloramphenicol resistant cells were selected to give strain UB-2131. Homologous recombination of the 3'-tails of these primers with the phage P22 genome caused the amplified DNA to replace P22 bps 40205-40800 with the CamR resistance cassette (coordinates according to GenBank entry BK000583). The CamR cassette region was then amplified from the UB-2131 P22 prophage with several hundred bp of P22 sequence on both sides and the resulting DNA was used to recombinationally replace the kanamycin resistant cassette *15^–^*Δsc302::KanR in the P22 prophage of UB-1790 (Padilla-Meier *et al.*, 2012). The resulting chloramphenicol resistant, kanamycin sensitive strain was called UB-2142 (its plaque-forming prophage is P22 UC-0938). To place the *c1*^7^ clear plaque mutation (Levine and Curtiss, 1961) into the prophage of UB-2142, we first sequenced the *c1* gene from *c1*^7^ mutant P22 *c1*^7^, *13^–^am*H101 (phage UC-0011) and found that it carries one coding difference from the wild type gene, a G to T change at P22 bp position 32189 that alters the *c1* gene glycine GGG codon 52 to a tryptophan TGG codon. We then replaced the UB-2142 prophage’s *c1* gene with a *galK* gene as follows: The *galK* expression cassette was amplified with primers whose 3'-tails allow homologous replacement of P22 DNA between bp 31865 and 32974 (a region that includes the 3'-portion of the P22 *c1* gene, orf48 and the 5'-part of gene *18*, none of which are required for prophage maintenence). Electroporation with this DNA and selection for *gal^+^* cells resulted in a prophage in which *galK* replaces these P22 bps (strain UB-2280). About 1 kbp of DNA containing the *c1^7^* mutation (as well as orf48 and the missing part of gene *18*) was amplified from phage P22 UC-0011 (above) and used to recombinationally replace the *galK* gene in the prophage of UB-2280, resulting in *Salmonella* strain UB-2158 whose prophage was named P22 UC-0937. A kanamycin resistant version of phage UC-0937 was also made by recombinationally neatly replacing the UB-2158 CamR cassette with a KanR cassette amplified from UB-1790 DNA and selection for kanamycin resistance. This is strain UB-2185 and its prophage is phage P22 UC-0944.

The P22 UC-0937 prophage in strain UB-2158 was characterized as follows: (i) Mitomycin C induction of UB-2158 growing in LB broth at a cell density of 2x10^8^/ml resulted in the release of about 10^11^ phage/ml (a phage yield similar to that of a *c^+^* P22 lysogen) that made clear plaques on UB-0002 but did not form plaques on UB-0001 (*i.e.*, it carries a clear mutation and an *amber* mutation in an essential gene). The induced lysogen lysed only after shaking with chloroform as expected of *13^–^* phage infections. (ii) Addition of tailspike protein did not increase the infectivity of phage produced after Mitomycin C induction of UB-2158, indicating that *sieA*^–^Δ1 allows good tailspike synthesis under these conditions and phage particles produced have the necessary complement of tailspikes. (iii) As expected, P22 UC-0937 is able to lysogenize *S. enterica,* but at a much lower frequency than *c^+^* phages; in an MOI=5 infection P22 UC-0937 formed chloramphenicol resistant lysogens on host UB-0002 about 10^–5^ as frequently as a parallel *c^+^* phage infection with P22 UC-0938. (iv) As expected, once formed, UC-0937 lysogens are stable and mitomycin C inducible (data not shown). (v) SDS polyacrylamide gel electrophoresis of purified P22 UC-937 virions showed the same protein bands as wild type P22 (data not shown). Finally, (vi) whole genome ILLUMINA sequencing of P22 UC-0937 showed that it contains the *sieA*^–^Δ1 deletion (Padilla-Meier *et al.*, 2012), the *c1*^7^ mutation (above), the *13^–^am*H101 mutation (Rennell and Poteete, 1985), and the orf25::CamR-EG1 chloramphenicol resistance cassette. Not surprisingly, because of its complex history that involved crosses between various P22 strains that had long independent laboratory histories, some of which may not have been backcrossed after mutagenesis (Botstein *et al.*, 1972), the complete sequence of P22 UC-0937 showed that it also carries seven other point differences from the reported P22 sequence (Accession No. BK000583 bp coordinates) as follows: synonymous nucleotide changes G1658A in codon 559 of gene *1*, C35869T change in codon 14 of *ninF* and A39314G in codon 109 of gene *15*, an insertion of an A between bps C41200 and C41201 in the *orf80* gene fragment created by the chloramphenicol resistance cassette insertion, and nonsynonymous changes C20549T (Glu33Lys) in *gtrB*, C35023A (His92Asn) in *ninB*, G39142A (Arg52Gln) in gene *15*. Since P22-UC-0937 does not require citrate for efficient plaque formation, this mutant gene 15 is functional, and none of the other differences is expected to have an impact on lysogeny or lytic growth of the phage.

**Figure S3. Predicted membrane topology of the three SieA sequence types.**

Membrane topologies of the three SieA protein types predicted by TMHMM (Krogh *et al.*, 2001). Note that types B and C have an N-terminal membrane spanning region that is not present in type A.

References

Adams, M., Brown, H., and Casjens, S. 1985. Bacteriophage P22 tail protein gene expression. *J. Virol.* 53, 180-184.

Botstein, D., Chan, R. K., Waddell, C. H., 1972. Genetics of bacteriophage P22. II. Gene order and gene function. Virology 49, 268-82.

Campoy, S., Hervas, A., Busquets, N., Erill, I., Teixido, L., Barbe, J., 2006. Induction of the SOS response by bacteriophage lytic development in *Salmonella enterica*. Virology 351**,** 360-7.

Casjens, S., Winn-Stapley, D., Gilcrease, E., Moreno, R., Kühlewein, C., Chua, J. E., Manning, P. A., Inwood, W., Clark, A. J., 2004. The chromosome of *Shigella flexneri* bacteriophage Sf6: complete nucleotide sequence, genetic mosaicism, and DNA packaging. J. Mol. Biol. 339**,** 379-394.

Ho, N., Lingohr, E., Villegas, A., Cole, L., Kropinski, A., 2012. Genomic characterization of two new *Salmonella* bacteriophages: vB_SosS_Oslo and vB_SemP_Emek. Ann. Agrar. Sci. 10**,** 18-23.

Jin, Q., Yuan, Z., Xu, J., Wang, Y., Shen, Y., Lu, W., Wang, J., Liu, H., Yang, J., Yang, F., Zhang, X., Zhang, J., Yang, G., Wu, H., Qu, D., Dong, J., Sun, L., Xue, Y., Zhao, A., Gao, Y., Zhu, J., Kan, B., Ding, K., Chen, S., Cheng, H., Yao, Z., He, B., Chen, R., Ma, D., Qiang, B., Wen, Y., Hou, Y., Yu, J., 2002. Genome sequence of *Shigella flexneri* 2a: insights into pathogenicity through comparison with genomes of *Escherichia coli* K12 and O157. Nucleic Acids Res. 30**,** 4432-41.

Johnson, T. J., Kariyawasam, S., Wannemuehler, Y., Mangiamele, P., Johnson, S. J., Doetkott, C., Skyberg, J. A., Lynne, A. M., Johnson, J. R., Nolan, L. K., 2007. The genome sequence of avian pathogenic *Escherichia coli* strain O1:K1:H7 shares strong similarities with human extraintestinal pathogenic *E. coli* genomes. J Bacteriol 189**,** 3228-36.

King, M. R., Vimr, R. P., Steenbergen, S. M., Spanjaard, L., Plunkett, G., 3rd, Blattner, F. R., Vimr, E. R., 2007. *Escherichia coli* K1-specific bacteriophage CUS-3 distribution and function in phase-variable capsular polysialic acid O acetylation. J Bacteriol 189**,** 6447-56.

Krogh, A., Larsson, B., von Heijne, G., Sonnhammer, E. L., 2001. Predicting transmembrane protein topology with a hidden Markov model: application to complete genomes. J Mol Biol 305**,** 567-80.

Levine, M., Curtiss, R., 1961. Genetic fine structure of the C region and the linkage map of phage P22. Genetics 46**,** 1573-80.

Owen, S. V., Wenner, N., Canals, R., Makumi, A., Hammarlof, D. L., Gordon, M. A., Aertsen, A., Feasey, N. A., Hinton, J. C., 2017. Characterization of the prophage repertoire of African *Salmonella Typhimurium* ST313 reveals high levels of spontaneous induction of novel phage BTP1. Front Microbiol 8**,** 235.

Pedulla, M. L., Ford, M. E., Karthikeyan, T., Houtz, J. M., Hendrix, R. W., Hatfull, G. F., Poteete, A. R., Gilcrease, E. B., Winn-Stapley, D. A., Casjens, S. R., 2003. Corrected sequence of the bacteriophage P22 genome. J Bacteriol 185**,** 1475-7.
